# Pearls and pointers on finger tip replants

**DOI:** 10.1186/1753-6561-9-S3-A55

**Published:** 2015-05-19

**Authors:** Hari Venkatramani

**Affiliations:** 1 Ganga Hospital, Coimbatore, 641043, India

## 

We have to be convinced that it is possible and worth the effort as it gives the best cosmetic and functional result.

Knowledge of the fingertip anatomy is very important , veins need at least 5 mm of dorsal skin proximal to nail fold. At the lunula level there is only palmar vein available.

Classification of amputation are many but Tamai's classification is still very good as it divides the finger tip at the level of lunula ie the same level where the digital arteries meet and form an arch. Hence proximal level will need digital artery repair and distal level will need central artery repair.

Finding the vessels: Veins are just under the dorsal skin, thus to visualise them, take the amputated part under the microscope and gently squeeze the pulp and a droplet of blood would come out of the vein. Skin flaps are raised with incisions kept in the centre. Thin full thickness flaps are raised.

Once the artery, nerve and vein have been identified they need to be tagged with 10-0 suture. It is good to excise all the fat around the vessels which will facilitate ease of clamp application. It is also beneficial to accommodate post operative oedema and primary closure

In distal Tamai Type I through nail or nail fold , we prefer to avoid a K-wire , here we stabilise the tip with only a secure nailbed repair. The advantage of this technique is that it facilitates small double clamp application and it also allows venous drainage along the medullary cavity of distal phalanx. We begin closing the skin from the dorsal to palmar side.

When palmar vein has to be done we prefer to do the artery anastomosis first as it is deeper. Palmar vein is done first followed by digital nerve and finally the artery.

We always do a vein first, if a vein is not found, we carry out arterial anastomosis first and after a few minutes the vein will fill up. If small gap is present, a vein graft is used by harvesting from the volar aspect of wrist. If veins are not done then chemical leeching is our preferred choice in which we make a slit in the tip and put a pledget soaked in heparin.

Nerve repair is always done, although in very distal tip, sensory recovery is still near

Normal in-spite of no formal nerve repair

Post-operatively a long arm cast is applied and only removed on day 10 under a brachial block and mobilisation started. All patients are given intravenous heparin 5000 units in 500ml of saline over 24 hours.

